# Disseminated Histoplasmosis Diagnosed in an Immunocompetent Patient from a Non-Endemic Area: Neglected or Emerging Disease?

**DOI:** 10.3390/diagnostics14192219

**Published:** 2024-10-05

**Authors:** Irina Ciortescu, Roxana Nemteanu, Ilinca Maria Chiriac, Silvia Zaharia, Alexandru Ionut Coseru, Diana Lacramioara Dumitrascu, Alin Vasilescu, Mihai Danciu, Catalina Ochisor, Alina Plesa

**Affiliations:** 1 Institute of Gastroenterology and Hepatology, “Saint Spiridon” University Hospital, 700111 Iasi, Romania; irinaciortescu@yahoo.com (I.C.); drsilviazaharia@gmail.com (S.Z.); ionutz_ionutz_barlad@yahoo.com (A.I.C.); alinaplesaro@yahoo.com (A.P.); 2 Medical Department, University of Medicine and Pharmacy “Grigore. T. Popa”, 700115 Iasi, Romania; ddianalacramioara@gmail.com (D.L.D.); alin.vasliescu@umfiasi.ro (A.V.); mihaida2@yahoo.com (M.D.); 3 Cardiology Department, “Saint Spiridon” University Hospital, 700111 Iasi, Romania; ilincamaria.chiriac@gmail.com; 4 Radiology Department, “Saint Spiridon” University Hospital, 700111 Iasi, Romania; 5 Surgical Department, “Saint Spiridon” University Hospital, 700111 Iasi, Romania; 6 Pathology Department, “Saint Spiridon” University Hospital, 700111 Iasi, Romania; 7 Infectious Disease Department, “Saint Spiridon” University Hospital, 700111 Iasi, Romania; ocatalina2004@yahoo.com

**Keywords:** *Histoplasma capsulatum*, histoplasmosis, disseminated histoplasmosis

## Abstract

*Histoplasma capsulatum* (*H. capsulatum*) is considered to be one of the most extensively spread dysmorphic fungi worldwide. Histoplasmosis primarily impacts patients with weakened immune systems and can result in a diverse range of clinical manifestations. In immunocompetent patients, the disease may manifest as a self-limiting or asymptomatic infection; however, in immunocompromised individuals, it can occur as a debilitating, disseminated disease. Diagnosing histoplasmosis may be challenging. A medical professional that specializes in treating endemic fungal illnesses is better able to assist with an accurate and timely diagnosis since they have a deeper grasp of these illnesses. Consequently, the process of diagnosing histoplasmosis might be difficult for less experienced physicians. The case presented is an example of the myriad faces that histoplasmosis can take on, mimicking other common infectious or malignant conditions, leading to extensive work-up and invasive procedures in establishing the diagnosis of this otherwise benign condition. We hereby report the case of disseminated histoplasmosis in a young immunocompetent female patient.

We present the case of a young 22-year-old female patient who came to the Emergency Department with a 3-week history of malaise, fever, abdominal pain, weight loss, and nausea. Her medical history was unremarkable. She denies drinking, smoking, or taking any medication. Upon questioning, she reports working in a poultry meat factory in Belgium, within the meat processing section (chicken meat manipulation for sausage manufacturing). She denies having any direct contact with the live animals. Physical examination at the time of admission revealed a high fever (39.5 °C), hepatosplenomegaly, and signs of dehydration. An extensive workup was performed to determine the cause of her fever and liver injury. Laboratory examination revealed mild anemia, elevated liver enzymes, cholestasis, and increased inflammatory makers.

Urine culture came back positive for *Escherichia coli*, while blood cultures remained negative. Viral hepatitis and HIV infection were suspected and ruled out by specific tests. The abdominal ultrasound showed hepatosplenomegaly with multiple hypoechoic nodules and enlarged lymph nodes. Computed tomography (CT) scans of the thorax and abdomen showed bilateral pulmonary nodules less than 3.5 mm in diameter localized in the superior lobes, as well as hepatomegaly with multiple hypodense nodules ranging from 3 to 23 mm in diameter, the largest one being 23 mm, localized in the third liver segment. Similar lesions were detected in the enlarged spleen, the largest one being 22 mm in diameter (Figure 1). In addition, lymph node enlargement and thickening of the wall of the lesser curvature of the stomach were detected. The patient was assessed for tuberculosis and syphilis by appropriate screening panel with negative test results.

Biopsy samples from both the antrum and corpus were taken during endoscopy, and the histopathology report confirmed the presence of *Helicobacter pylori* infection and chronic gastritis. A heart ultrasound was performed to rule out any heart and valvular abnormalities. Considering the clinical presentation and biological and imaging data, the differential diagnosis included non-Hodgkin’s lymphoma, systemic bacterial infection, and systemic histoplasmosis.

Consequently, a laparoscopic biopsy (Figure 2) of the liver and lymph nodes was performed. The histopathological examination and special PAS staining reported a positive diagnosis for the presence of *H. capsulatum* (Figure 3).

Serological testing for histoplasmosis came back negative. Antibiotics were initiated to treat her urinary tract infection. The patient was referred to the Infectious Disease Clinic and received treatment with itraconazole orally for seven days. Subsequently, due to the patient’s persistent fever, a therapeutic switch to posaconazole (300 mg loading dose twice per day on the first day, after which 300 mg/day) was indicated, resulting in a favorable clinical and biological outcome. The patient was recommended completing a one-year course of oral treatment with itraconazole and routine follow-up.

Histoplasmosis is a non-contagious fungal infection distributed worldwide caused by *Histoplasma capsulatum* (*H. capsulatum*) [1]. Since its first description in 1906 by the pathologist Samuel Darling, the perception of the disease has shifted from a rare, acute, and lethal disease to an asymptomatic benign form, rarely recognized clinically by physicians and often misdiagnosed as tuberculosis [2]. The pathogen is capable of infecting both human and animal hosts. The spores can travel through air ventilation systems, be mixed in with fertilizers, or distributed by wild and domestic animals [3]. Human-to-human transmission is unlikely (with the exception of organ transplant recipients), yet fungal acquisition is possible via the respiratory tract after inhalation of small fragments of saprophytic spores [4]. Within the alveoli, the antigen undergoes a transformation into a yeast form either within or outside of phagocytes. Host phagocytes are pivotal in the pathogenesis of histoplasmosis, serving as the primary carriers for the fungal dissemination, which begins in the lymph nodes and subsequently spreads to various organs [5].

A shift in the epidemiological trends was observed during the HIV pandemic, which was linked to an increase in histoplasmosis cases globally, emerging especially from non-endemic areas [6,7]. In a recent systematic review from 2024 assessing a study cohort of 109 cases, Kontogiannis et al. reported a significant increase in cases of histoplasmosis in Italy, a non-endemic area, and Belgium. Interestingly, no patients from Romania were detected [8]. In the case presented, travelling from Belgium and having exposure to animals and meat products may be considered potential risk factors for infection acquisition.

Most documented cases of disseminated histoplasmosis occur in immune-compromised individuals, such as those with HIV infection [9]. Disseminated histoplasmosis in an otherwise healthy patient is an exceedingly uncommon occurrence. The majority of healthy people who are exposed to *H. capsulatum* conidia will remain asymptomatic, while others will develop flu-like symptoms or even severe life-threatening complications [10,11]. The digestive tract is frequently affected, and liver involvement in association with splenomegaly, lymphadenopathy, and pancreatic involvement are well-known features [12]. One explanation for the disseminated form of histoplasmosis diagnosed in our young healthy patient may be prolonged exposure to a high quantity of inoculum [10].

The most reliable methods of diagnosis include culture-based isolation of *H. capsulatum* or histopathological detection of yeast. Additionally, practitioners have access to serology and antigen testing, the latter of which is both very sensitive and easily interpretable. The incubation period for this fungus is 7 to 3–17 days for its acute form [13]. In the case presented, the diagnosis was made through targeted biopsy samples identifying the infectious antigen. The predominant pattern is represented by granulomatous caseating or noncaseating nodules, but this is not specific for histoplasmosis [14]. In patients with disseminated histoplasmosis, yeast-like structures inside circulating phagocytic cells on a routine peripheral blood smear can be seen [15].

Antigen and antibody detections in urine and serum can be utilized for the rapid diagnosis of histoplasmosis, but these methods are still unavailable to all centers. Specific antibodies are of limited significance in the diagnosis of acute histoplasmosis because they manifest within a 2- to 6-week period following exposure [16]. However, they are more valuable in chronic cases of histoplasmosis. Interestingly, the sensitivity of these tests is higher in disseminated histoplasmosis in whom the burden of infection is substantial, and antigenuria can be detected in the majority of cases [17]. In our case, the antibody levels were assessed, and a negative result was obtained. Urinary antigen detection was not possible in our hospital.

Treatment options include two different types of antifungal agents: the triazoles (like fluconazole, itraconazole, voriconazole, posaconazole, and isavuconazole) and the polyenes (amphotericin B conventional or liposomal formula) [18,19]. Itraconazole is the drug of choice for the treatment of the various forms of histoplasmosis as a sole therapy or as a step-down therapy following amphotericin B infusion [20]. The primary objectives for treatment include acute pulmonary histoplasmosis, disseminated illness, and histoplasmosis in immunocompromised persons. Asymptomatic cases do not benefit from treatment [20,21]. Itraconazole can be used for the treatment of milder symptomatic forms and to prevent relapse, while intravenous amphotericin B is reserved for treatment of moderate or severe disease [21]. In the case presented, the physician opted for agents from the triazoles class, which proved effective in managing the infection.

To summarize, in cases of disseminated histoplasmosis, a diagnostic algorithm may be employed: when confronted with isolated community-acquired pneumonia of unknown etiology unresponsive to antibiotics or accompanied by hepatosplenomegaly, lymphadenopathy, and pancreatic involvement; significant exposure to bird or bat droppings; imaging studies revealing pulmonary nodules or lymphadenopathy; and a connection to a recognized histoplasmosis outbreak, histoplasmosis should be considered a potential diagnosis. An enzyme immunoassay urine antigen test should be utilized as the initial screening tool, followed by antibody detection by immunodiffusion, complement fixation, or culture to confirm the diagnosis. It should be kept in mind that *Histoplasma* are a frequent but likely underreported cause of fungal infection present worldwide in pockets of endemicity. As suspected, climate change and unrestricted travelling to endemic regions have altered the patterns of distribution for *H. capsulatum*, enabling disease occurrence in unexpected regions.

In conclusion, the clinical presentation of histoplasmosis is non-specific and can easily be mistaken for other conditions. Managing patients with histoplasmosis is challenging because its symptoms resemble those of other illnesses, including malignancies. This can lead to misdiagnosis, higher morbidity rates, and increased economic burden. To improve the diagnosis and treatment of histoplasmosis, it is important to raise awareness through clinical trials and review studies. Clinical suspicion remains of great importance for an early diagnosis of histoplasmosis, particularly when assessing cases from a nonendemic area.

## Figures and Tables

**Figure 1:**
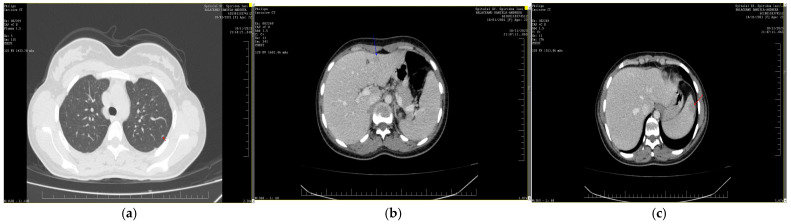
CT scans from thorax (**a**), liver (**b**), and spleen (**c**) showing multiple hypoechoic, circumscribed, randomly distributed nodules situated in both lungs, liver, and spleen and enlarged lymph nodes.

**Figure 2:**
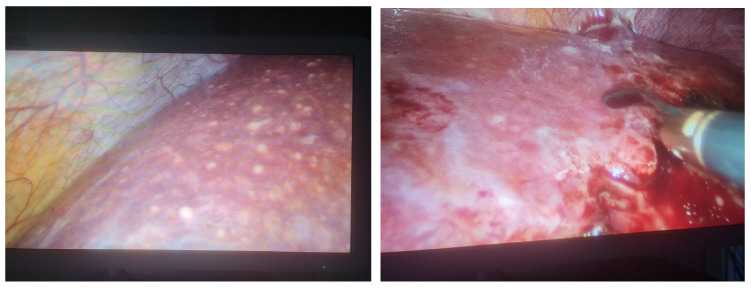
Laparoscopic view (2× magnification) of liver appearance and biopsy sampling of liver tissue that is infiltrated by small yellowish nodules.

**Figure 3:**
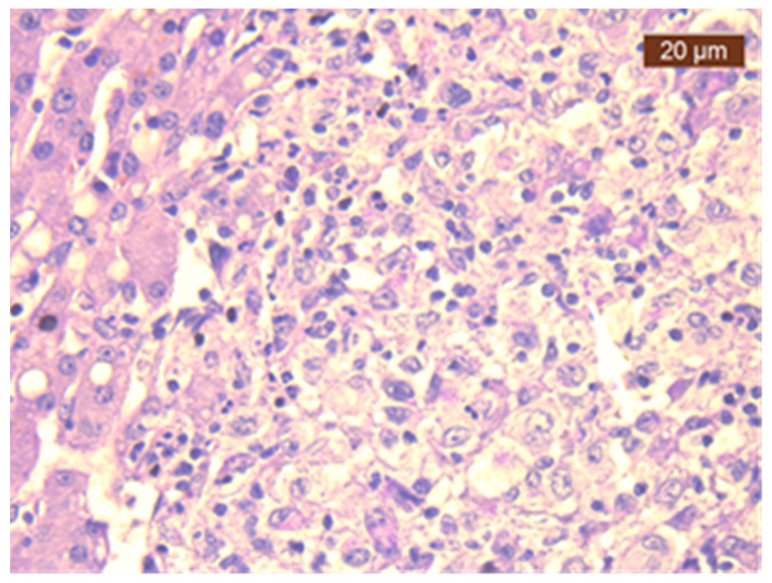
Liver biopsy showing epithelioid granulomas with dot-like intracytoplasmic *Histoplasma* (PAS staining, 400× magnification).

## Data Availability

The original contributions presented in the study are included in the article, further inquiries can be directed to the corresponding author.
